# Regulatory non-coding RNA: new instruments in the orchestration of cell death

**DOI:** 10.1038/cddis.2016.210

**Published:** 2016-08-11

**Authors:** Ye Su, Haijiang Wu, Alexander Pavlosky, Ling-Lin Zou, Xinna Deng, Zhu-Xu Zhang, Anthony M Jevnikar

**Affiliations:** 1 Matthew Mailing Centre for Translational Transplantation Studies, Lawson Health Research Institute, London Health Sciences Centre, University of Western Ontario, London, Ontario, Canada; 2 Department of Medicine, University of Western Ontario, London, Ontario, Canada; 3 Department of Pathology, University of Western Ontario, London, Ontario, Canada; 4 Key Laboratory of Kidney Diseases, Department of Pathology, Hebei Medical University, Shijiazhuang, China; 5 Department of Oncology, Affiliated Hospital of Southwest Medical University, Luzhou, Sichuan, China; 6 Department of Oncology and Immunotherapy, Hebei General Hospital, Shijiazhuang, China

## Abstract

Non-coding RNA (ncRNA) comprises a substantial portion of primary transcripts that are generated by genomic transcription, but are not translated into protein. The possible functions of these once considered ‘junk' molecules have incited considerable interest and new insights have emerged. The two major members of ncRNAs, namely micro RNA (miRNA) and long non-coding RNA (lncRNA), have important regulatory roles in gene expression and many important physiological processes, which has recently been extended to programmed cell death. The previous paradigm of programmed cell death only by apoptosis has recently expanded to include modalities of regulated necrosis (RN), and particularly necroptosis. However, most research efforts in this field have been on protein regulators, leaving the role of ncRNAs largely unexplored. In this review, we discuss important findings concerning miRNAs and lncRNAs that modulate apoptosis and RN pathways, as well as the miRNA–lncRNA interactions that affect cell death regulation.

## Facts

- ncRNAs comprise a major part of poly-A tailed mature RNAs and are no longer considered ‘transcriptional noise' as they have key functions that do not involve translation.
- Both miRNA and lncRNA, either alone or in interaction with each other, extensively modulate the inter-related steps and mediators of programmed cell death.
- Current cell death networks are charted mostly on protein regulators, whereas ncRNAs constitute the ‘invisible' but intertwined part of these networks.
- ncRNA binding is mostly sequence/structure dependent, which makes some of their unexplored death-regulating activities, for example, the regulation of necroptosis by lncRNA, partially predictable by *in silico* bioinformatics.

## Open questions

- In other evolving forms of programmed cell death such as ferroptosis, pyroptosis and autophagy, what is the role of ncRNA and how does it interact with established protein networks.
- Does a newly proposed sequence-dependent competition model between mRNAs and lncRNAs with miRNAs apply to cell death regulation?
- Can necroptosis-related lncRNAs predicted *in silico* be validated in experimental models and applied to therapeutics for inflammation?

**Figure 1:**
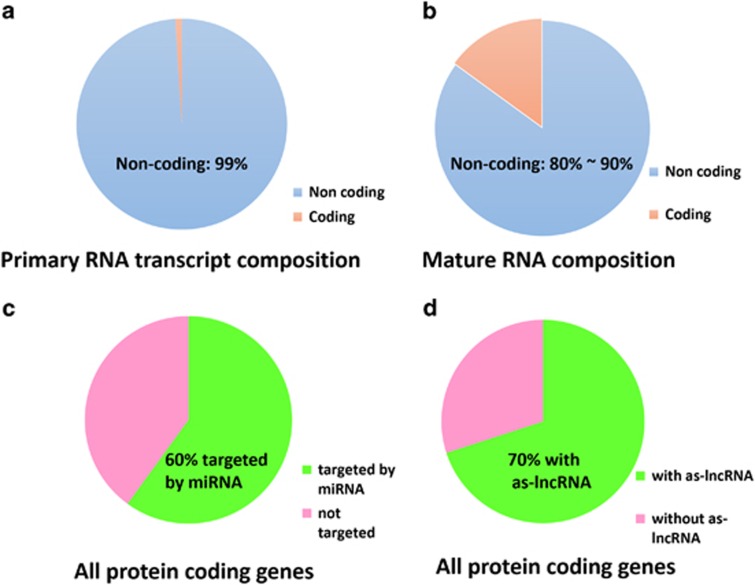
The biological significance of ncRNAs. (**a**) ncRNAs comprise 99% of primary RNA transcripts generated by the genomic transcription. (**b**) ncRNAs comprise 80 to 90% of the poly-A-tailed mature RNAs. (**c**) Over 60% of the protein-coding genes in human are targets of miRNA. (**d**) About 70% of the protein-coding genes have at least one homologous antisense (as)-lncRNA

Study of the human genome, which contains over 3 billion base pairs, has revealed that relatively few transcripts lead to productive protein translation. Specifically, primary transcripts for as much as 93% of genomic sequences^1^ were identified in the cytoplasm by the Encyclopedia of DNA Elements (ENCODE) Project, highlighting that a mere 1% undergo protein encoding (Figure 1a). Poly-adenylated (poly-A) tails are a hallmark of mature RNAs, which function to stabilize the RNA and facilitate its export from the nucleus. However, poly-A tails are present in not only mature mRNAs, but also many non-coding RNAs (ncRNA) of either intermediary^2, 3^ or mature forms.^4^ The cytosolic poly-A RNAs represent about 5–10% of the genome sequence,^5^ which still dwarfs the small 1% that account for protein encoding. Therefore, the vast majority of poly-A RNAs are indeed ncRNAs (Figure 1b).

Traditionally, ncRNAs have been arbitrarily categorized into long non-coding RNAs (lncRNAs), which are longer than 200nt, and small ncRNAs (sncRNAs), which are shorter than 200nt. The latter can be further subdivided into various categories, including micro RNAs (miRNAs), piwiRNAs (piRNAs) and small nuclear RNAs (snoRNAs).^6^ Although these may collectively or individually alter cell death, this review will focus on the two most important ncRNAs currently identified in cell death regulation: miRNA and lncRNA.

## miRNA and programmed cell death

### Mechanisms of miRNA-mediated gene expression regulation

The miRBase (version 21.0) confirms that 28 645 miRNA transcripts from 206 species, including 2661 human miRNA transcripts,^7^ regulate over 60% of human genes^8^ (Figure 1c). miRNAs typically bind to the 3'-untranslated region (UTR)^9^ of protein-coding mRNA by imperfect sequence-specific recognition^9, 10^ to either degrade it^11, 12, 13^ or repress its translation.^13^ As such, mechanisms such as the alternative cleavage and polyadenylation that generates different 3'-UTR isoforms affect the miRNA targeting efficiency,^14^ whereas the translation suppression is dependent on the miRNA-induced silencing complex (miRISC) and the CCR4-NOT complex, which recruits and locks the eIF4A2 on the mRNA region between the pre-initiation complex and the start codon. The eIF4A2 then serves as a roadblock that prevents the former from scanning along the mRNA strand and reaching the latter.^15, 16, 17^ CCR4-NOT also uses its subunit CNOT1 to recruit DDX6 as a downstream factor, therefore disrupting CNOT1–DDX6 interaction abrogates miRNA repression.^18^ Those mRNAs with regulatory AU-rich elements (AREs) in their 3'-UTR can be bound by HuR, which dissociates miRISC and therefore relieves miRNA repression.^19^ In addition, competing endogenous RNAs (ceRNAs), such as lncRNA (will be discussed later) and cirRNA,^20, 21^ sequester miRNAs and therefore prevent them from binding and repressing target mRNAs. Some miRNAs, like miR-Let-7 and miR369-3, which normally suppress translation, can activate translation under certain situations such as cell cycle arrest, or in cooperation with some transcription factors.^22^ miRNAs can bind to mRNA 5'-UTR as well,^23^ which mostly activates transcription, although suppression has also been reported.^24, 25^

### miRNA and intrinsic apoptosis

#### Mediators of intrinsic apoptosis

Although the term ‘intrinsic apoptosis' typically refers to mitochondrial-centered apoptosis pathways, other organelles, like ER,^26, 27, 28, 29^ lysosomes^30, 31, 32, 33, 34^ and Golgi apparatus,^35, 36^ also participate in apoptosis. The term ‘intrinsic apoptosis' used in this review, unless otherwise specified, denotes mitochondrial-related apoptosis. Initiation of intrinsic apoptosis essentially relies on three categories of B-cell lymphoma 2 (BCL2) family members, namely the pro-apoptotic members: Bcl-2-associated X protein (BAX) and Bcl-2 homologous antagonist/killer (BAK), the anti-apoptotic members: BCL2, MCL1, BCL-XL, etc., and the BCL2 homology domain 3 (BH3)-only proteins: Bim, Bid, Puma, Bad, etc. Perturbation of the dynamic balance between counter-acting members leads to oligomerization of BAX and BAK on the outer membrane of mitochondria and the mitochondria outer membrane permeabilization (MOMP), thus initiating cytochrome-c release into cytosol. Cytosolic cytochrome-c binds Apaf-1 (ref. 37) to facilitate the formation of the multi-protein complex known as the apoptosome.^38, 39^ This process is negatively regulated by heat shock protein 70 (HSP70), which is typically seen during cellular stress.^40^ The apoptosome activates caspase-9 and the downstream caspase cascade.^41^ Caspase-3, which is located at the convergence of intrinsic and extrinsic apoptosis that initiates the ultimate apoptotic executioner mechanisms, is sequentially activated by caspase-9. Caspase-9 can also be cleaved and activated by caspase-3 as a positive feedback control.

#### miRNAs work on BCL2 to regulate intrinsic apoptosis

**Figure 2:**
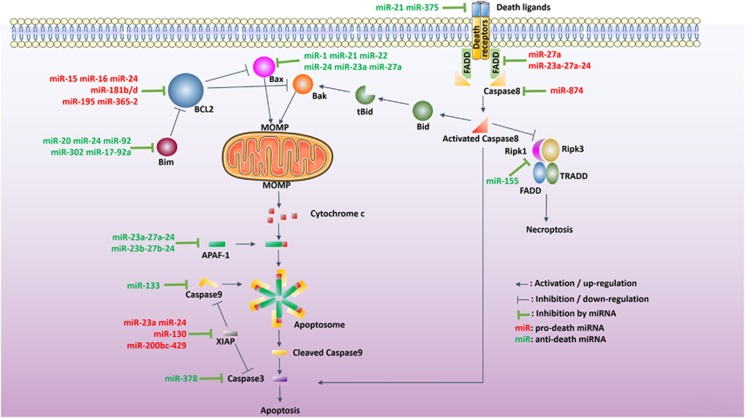
miRNAs that regulate cell death pathways. miRNAs could either promote (in red) or inhibit (in green) cell death by regulating key mediators of intrinsic apoptosis, extrinsic apoptosis and necroptosis

miRNAs are profoundly involved in cell death regulation, as the deletion of the miRNA-processing RNase III enzyme Dicer^42, 43^ in neural stem cells^44^ and neural crest derivative cells^45^ leads to pervasive cell death. Other miRNA processors, such as Drosha, DGCR8 and XPO5, also profoundly affect cell death.^46, 47^ Specifically, numerous miRNAs regulate BCL2, a key mediator of the intrinsic apoptosis pathway (Figure 2 and Table 1) that is overexpressed in certain pathological situations. In B-cell malignancies such as chronic lymphocytic leukemia (CLL), the overexpression of BCL2 is concomitant with the marked downregulation of two miRNAs: miR-15 and miR-16, both induce apoptosis by suppressing BCL2 when ectopically expressed.^48^ The typical binding sites of numerous BCL2-inhbiting mRNAs, such as miR-195 miR-24 and miR-365-2, are within the 3'-UTR. Overexpression of these miRNAs facilitate apoptosis in otherwise apoptosis-resistant breast cancer MCF7 cells.^49^ Especially, miR-195 triggers apoptosis through free fatty acids and thus may have therapeutic potential in lipotoxic cardiomyopathy.^50^

As the overexpression of BCL2 is one of the major contributing factors to the development of multidrug resistance (MDR, a mechanism by which cancer cells resist structurally and mechanistically unrelated chemotherapeutic drugs), BCL2-specific miRNAs can also be used to treat MDR. For example, miR-181b that reduces BCL2 expression overcomes MDR by facilitating apoptosis in several MDR cancer cell lines.^51^ miR-181d also targets BCL2 to induce apoptosis and cell cycle arrest.^52^ Refractive B-cell MDR malignancy can be treated by inducing miR-125b and miR-155-mediated BCL2 suppression.^53^ BCL2-specific miRNAs also induce apoptosis in other cancer cells, whether MDR or not, for example, miR-125b in hepatocellular carcinoma (HCC) cells,^54^ miR-7 in the non-small cell lung cancer cell line A549 cells^55^ and miR-497 in gastric and lung cancer cell lines.^56^

Physiological or pathological perturbations both inside and outside cells often influence the expression of miRNAs, which could in turn precipitate intrinsic apoptosis. For instance, in heart ischemia–reperfusion injury (IRI), fluctuations of miR-1, miR-21, miR-29, miR-92a, miR-133, miR-199a and miR-320 levels change the expression of many miRNA-regulated genes, including phosphoinositide 3-kinase, phosphatase and tensin homolog deleted on chromosome 10 (PTEN), Bcl-2, Mcl-1, HSP60, HSP70, HSP20, programmed cell death 4 (Pdcd4), LRRFIP1, Sirt-1, etc., which can individually or collectively trigger intrinsic apoptosis and thus affect the severity and manifest of injury.^57^

#### miRNAs directly or indirectly affect BAX to modulate intrinsic apoptosis

miRNAs also work on BAX. This pro-apoptotic BCL2 family protein is mainly found in the cytosol and undergoes conformational changes upon apoptosis induction^58^ by associating with the mitochondrial membrane^59^ to mediate the formation of MOMP.^58, 60^ Ischemic preconditioning, a strategy that increases heart tissue resistance to subsequent IRI, leads to downregulation of BAX, and upregulation of miR-1 and miR-21, indicating both may target BAX to inhibit apoptosis and confer resistance to further cardiac IRI.^61^ Viral vectors can be used to deliver miRNA against BAX. For example, the adenovirus-mediated miR-22 overexpression indirectly inhibits BAX by working on CREB-binding protein.^62^ miRNAs can inhibit BAX not only at the expression level, as miR-24 has been shown to suppress the translocation of BAX from the cytosol to the mitochondrial membrane, thus inhibiting cytochrome-c release and execution of apoptosis.^63^ Similar protective effects of miRNAs were also observed in the central nervous system. miR-23a and miR-27a were rapidly downregulated in the first hour following traumatic brain injury, and their ‘mimetics' effectively inhibited neuronal cell apoptosis to limit the cortical lesion volume.^64^

#### miRNAs modulate intrinsic apoptosis by affecting Bim or Bim-related mechanisms

Other than being directly regulated by miRNAs, as a typical BH3-only member, Bim contains a BH3 (ref. 65) with which it interacts with other members of the BCL2 family such as BCL2,^65^ BCL2-XL^65, 66^ and MCL1,^67, 68^ and integrates miRNAs regulation exerted on them. miR-20, miR-92 and miR-302 target and regulate Bim to maintain the low-apoptotic threshold for survival of mammalian primed pluripotent stem cells.^69^ MiR-24, as mentioned previously, also directly binds to the 3'-UTR of Bim to suppress it. After mouse acute myocardial infarction, miR-24 is downregulated in the left ventricular ischemic border zone. Using lipofectamine-mediated transfection for *in vivo* local delivery to mouse hearts, ‘miR-24 mimics' inhibited apoptosis and reduced infarct size as well as cardiac dysfunction by specifically suppressing Bim.^70^ Interestingly, BAX translocation inhibition by miR-24 can be overcompensated for by Bim deletion. Upon Bim deletion, BAX translocation following miR-24 treatment is reduced below basal levels, indicating miR-24 indirectly inhibits BAX translocation – most likely by targeting Bim. Moreover, this overcompensation hints that Bim may exploit another apoptosis-regulating mechanism that is independent of miR-24.^63^ Bim-regulating miRNAs can also be involved in the apoptosis-regulating effects of some clinical reagents. For example, the miR-17-92a cluster binds Bim on 3'-UTR and suppresses it. Depletion of this cluster augmented dexamethasone-triggered apoptosis, whereas its overexpression facilitated the anti-apoptotic effects of estrogen on osteoblasts.^71^

#### miRNAs regulate downstream mechanisms of intrinsic apoptosis

MOMP and release of cytochrome-c, by causing mitochondria swelling and rupture, are traditionally believed to irreversibly commit the cell to death. Owing to the loss of oxidative phosphorylation, cell death is inevitable at this point and cannot be salvaged even by caspase inhibitors.^72^ However, some have suggested that the irreversible death commitment point cannot only be extended beyond cytochrome-c release,^73^ but also that the structural and functional integrity of the mitochondria can be restored after cytochrome-c release.^74^ Indeed several mechanisms downstream of cytochrome-c release are also reported to be available for anti-apoptotic intervention. As a pivotal component of the apoptosome and an essential intrinsic apoptosis mediator downstream of cytochrome-c release, apaf-1 is modulated by four miRNAs (miR-23a/b and miR-27a/b) that form two clusters, miR-23a-27a-24 and miR-23b-27b-24. As such, mouse neuronal-specific transgenic overexpression of miR-23b and miR-27b attenuated hypoxia-induced apoptosis.^75^ Furthermore, caspase-9 contains a miR-133-binding site in its 3'-UTR, and upregulation of miR-133 by ischemic post-conditioning protects rat hearts against ischemia–reperfusion-induced apoptosis.^76^ Caspase-3 is targeted by miR-378, as confirmed by luciferase reporter assay, and ‘miR-378 mimic' transfection and overexpression substantially suppressed apoptosis and enhanced cell viability in mouse myocardial ischemia. Conversely, miR-378 inhibitor aggravated hypoxia-induced apoptosis.^77^

Besides direct regulations, miRNAs also target caspase modulators for indirect regulation. X-linked inhibitor of apoptosis protein (XIAP),^78^ for example, binds and inhibits caspase-9 and caspase-3, and sequesters activated caspase-3 within the apoptosome complex to inhibit its function.^79^ Several miRNAs, namely miR-23a,^80^ miR-24,^81^ miR-130 (ref. 82) and miR-200bc-429 (ref. 83) cluster, target the XIAP 3'-UTR and therefore enhance apoptosis in various cell types.

### miRNA and extrinsic apoptosis

Extrinsic apoptosis is initiated by the coupling of membrane-bound death receptors (DRs) to cognate ligands. There are six known DRs of which TNF receptor (TNFR) and Fas have been most well characterized. The intracellular domains of activated death ligands recruit a number of adaptor proteins that relay the extrinsic signal into the caspase cascade, which starts with caspase-8 activation. The death ligands can be targeted by miRNAs. For example, the anti-apoptotic effect of miR-21 by targeting Fas ligand (FasL) was reported in hypoxic cardiomyocytes,^84^ neurons^85^ and hepatocytes.^86^ Interestingly, miR-21 is subject to positive regulation by Akt, which makes miR-21 a mediator of Akt-FasL regulation.^84^ Adaptor proteins immediately downstream of DRs are also targeted. For example, Fas-associated protein with death domain (FADD) is targeted by miR-27a on its 3'-UTR. Similarly, the miRNA cluster miR-23a-27a-24 could independently induce apoptosis, or enhance TNF*α*-induced apoptosis, by reducing FADD expression.^87^

miRNAs work further downstream the pathway as well. miR-375 enhances TNF*α*-induced apoptosis, the underlying mechanisms of which remains largely elusive, although reductions in both cIAP and cFLIP-L by miR-375 have been observed.^88^

### miRNA and programmed necrosis

Necrosis was traditionally considered as the consequence of severe, accidental, non-physiological stress, which results in un-regulated cell ‘explosion', and the release of pro-inflammatory cytoplasmic/nuclear contents. With the discovery of several modalities of regulated necrosis (RN), such as necroptosis, it has now been accepted that necrosis can also be triggered by programmed and often counter-balanced intracellular pathways. Necroptosis, for example, can be initiated by ligation of TNF-*α* to TNFR) when caspase-8 is inhibited.^89, 90^ It is mediated by the kinases RIPK1, RIPK3 (refs 89, 90) and the pseudo-kinase MLKL.^91^ MiR-155 targets RIPK1 and has been shown to be markedly upregulated following hydrogen-peroxide treatment in cardiomyocyte progenitor cells (CMPCs). Overexpression of miR-155 attenuates CMPC necrosis to a level similar to that achieved with the RIPK1 inhibitor necrostatin-1.^92^ miR-874, on the other hand, enhances necroptosis by targeting caspase-8 and abolishing its inhibition on RIPK1/RIPK3.^93, 94^ In addition, Foxo3a can repress miR-874 to reduce necroptosis.^95^ These findings indicate that miRNA may be important potential regulators of programmed necrosis with inhibitory effects that may be comparable to pharmacological intervention. This may be of considerable therapeutic significance with the current paucity of small molecules that can block specific pathways or RN.

## LncRNA and programmed cell death

### LncRNA classification

As lncRNAs are transcribed by RNA polymerase II before getting capped and poly-A, they can be categorized based on their relative positions to known neighboring ‘protein-encoding' exons. Those transcribed from between protein-encoding genes are known as long intergenic lncRNAs, which comprise the largest portion of lncRNAs, whereas those from intronic regions are called intronic lncRNAs.^96^ Antisense lncRNAs are transcribed from the opposite DNA strand as the protein transcript template and often overlap parts of mRNAs.^97, 98^ In all, 70% of mouse genes may have overlapping antisense lncRNA transcripts^99^ (Figure 1d). A mutated gene that has lost its protein production capability can also produce an lncRNA pseudogene.^100, 101^ All of the above mentioned lncRNAs epigenetically resemble protein-coding genes, in that they feature similar high histone 3 lysine 4 tri-methylation (H3K4me3) marks. In contrast, enhancer lncRNAs (eRNAs), which are transcribed from the intergenic enhancer regions, distinguish themselves by expressing histone 3 lysine 4 mono-methylation (H3K4me1) marks instead.^102, 103, 104, 105^

LncRNAs were once considered transcription ‘noise' partly because they lack enough sequence conservation, a hallmark of protein-coding genes. However, Johnsson *et al.*^106^ revealed that their secondary, rather than primary, structure could be evolutionarily conservative, and thus could serve as the main functional unit. In addition, tissue specificities of lncRNAs are also better preserved than primary sequences,^107^ hinting at the importance of conservative secondary structure.

### Mechanisms of lncRNA-mediated gene expression regulation

LncRNAs could potentially affect the expression of a vast majority of genes, as in the mouse genome, 70% of protein-coding genes have at least one homologous antisense lncRNA,^99^ which is not even the major subtype of lncRNA (Figure 1d). LncRNAs can regulate gene expression via several different mechanisms. First, they can directly act on the genomic DNA to regulate expression. Recent studies have established that some lncRNAs are either retained to their transcription sites (cis) or translocated to remote sites (trans)^108, 109, 110^ to recruit chromatin modifying complexes that dictate the formation of heterochromatin that represses transcription. Other lncRNAs activate transcription by either inducing 3-dimensional chromatin conformation changes^111, 112^ or triggering enhancer regions.^113^ Second, lncRNAs can interact with proteins, namely transcription factors and some RNA-binding proteins, to indirectly regulate transcription. In this manner, LncRNA acts as a decoy and sequesters transcription factors from binding to their DNA targets.^114, 115, 116^ LncRNA has also been reported to allosterically modify RNA-binding proteins that regulate transcription.^117^ In particular, the steroid receptor activator (SRA) lncRNA acts as a component of the steroid coactivator complex,^118, 119^ wherein it relies on its secondary structure^120^ to co-activate other transcription factors,^121^ which again underlines the importance of secondary structure in lncRNA function. As an lncRNA, SRA lncRNA is somewhat eccentric in that it actually encodes several short peptides by utilizing different promoters and splicing patterns. These peptides in turn bind to SRA lncRNA and inhibit it from co-activating other transcription factors, forming a negative feedback loop.^122, 123, 124^ Third, lncRNAs can indirectly alter gene expression by competing with miRNA as ceRNAs,^125^ an important regulatory RNA category comprises various ncRNAs and even some mRNAs with profound effects beyond cell death.^126^ More about the miRNA-centered ceRNA crosstalk is discussed in section LncRNAs interact with miRNA in regulating cell death – 'lncRNA–miRNA interaction'.

### LncRNAs regulate apoptosis on various levels

**Figure 3:**
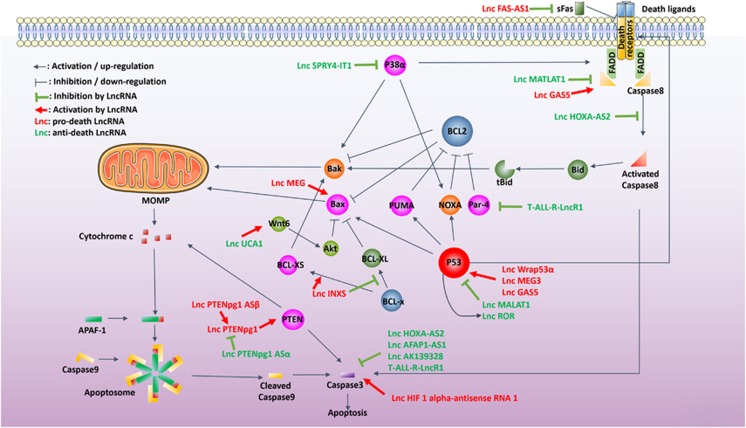
LncRNAs that regulate cell death pathways. LncRNAs could either promote (red) or inhibit (green) cell death by regulating key mediators of intrinsic apoptosis and extrinsic apoptosis

Numerous reports indicate that lncRNAs may modulate apoptosis on various levels and in different patterns (Figure 3 and Table 1). Many of these studies were conducted in cancer cell lines that augment their lncRNAs expression to evade apoptosis. The urothelial cancer-associated 1 (UCA1) lncRNA, which is highly expressed in human bladder cancer cells, upregulates wingless-type MMTV integration site family member 6 (Wnt6), therefore enhancing the inhibition of BAX by Akt, and conferring resistance to cisplatin-induced apoptosis.^127, 128^ In addition, T-ALL-R-LncR1, an lncRNA that is associated with T-cell acute lymphoblastic leukemia (T-ALL) and is abnormally expressed in some tumor tissues, suppresses caspase-3, inhibits Par-4 (which inhibits BCL-2 and NF-κB)^129^ induces apoptosis, and augments pro-apoptotic Smac protein expression.^130^ URHC (upregulated in HCC) lncRNA is highly expressed in HCC and inhibits the apoptosis-inducing sterile alpha motif and leucine zipper containing kinase AZK (ZAK) gene.^131^ Other apoptosis-suppressing lncRNAs expressed by cancer cell lines include: HOXA-AS2, which suppresses TRAIL expression and the cleavage of caspase-8, -9 and -3 in the promyelocytic leukemia cell line NB4;^132^ SPRY4-IT1, which is an inhibitor of MAPK pathway (MAPK pathway leads to p38*α* activation, upregulates BAX Bim Noxa Fas-FasL) in the melanoma cell line WM1552C;^133^ PlncRNA-1 in the prostate cancer cell line LNCaP,^134^ and AFAP1-AS1, which inhibits caspase-3 cleavage, in the esophageal adenocarcinoma cell line OE-33 (ref. 135) and the esophageal squamous cell carcinoma.^136^

In contrast, several lncRNAs have been shown to promote apoptosis and therefore mediate pathological injury or damage. AK139328, an lncRNA that is highly expressed in normal mouse liver, mediates apoptosis in liver IRI. Knockdown of AK139328 reduces caspase-3 activation and ameliorates injury.^137^ In vascular smooth muscle cells, the lncRNA HIF 1 alpha-antisense RNA 1, which is under positive regulation of Brahma-related gene 1, activates caspase-3 and promotes apoptosis and contributes to pathogenesis of thoracic aortic aneurysms.^138^ Aside from aberrant expression of anti-apoptotic lncRNAs, cancer cells could also downregulate pro-apoptotic lncRNAs to evade apoptosis. Growth arrest-specific 5 (GAS5) lncRNA, which effectively promotes apoptosis, is significantly downregulated in prostate cancer cells as they acquire resistance to apoptosis.^139^ Apoptosis induced by GAS5 is dependent on caspase-8 but not caspase-9,^140^ and may involve the upregulation of p53.^141^ In human HCC cells, the apoptosis-promoting uc002mbe.2 lncRNA is significantly lower compared with normal cells, but can be induced to be expressed 300-fold higher following treatment of Trichostatin A (TSA), a histone deacetylase inhibitor, and thus has a pivotal role in TSA's antitumor effect.^142, 143^

### The regulation of p53 by lncRNAs

p53 provides crucial upstream apoptosis control of both intrinsic and extrinsic pathways by directly regulating several key mediators such as BAX,^144^ NOXA,^145^ PUMA^146^ and Bid^147^ (which bridges the intrinsic and extrinsic pathway), all of which contain p53-responsive elements in their promoters.^148^ Besides its direct regulation of BAX, p53 also indirectly promotes mitochondrial translocation and polymerization of BAX by effectively regulating PUMA^149^ because of its significantly higher affinity to the PUMA promoter than to the BAX promoter.^150^ p53 also triggers the extrinsic apoptosis pathway as it induces cell surface DRs including Fas, TRAIL-R2 (DR5) and PERP, by promoting either mRNA expression (Fas, DR5, PERP),^151, 152, 153, 154^ or protein translocation from Golgi apparatus to cell surface (Fas),^155^ all of which lead to caspase-8 activation.

p53 may itself be subject to sophisticated regulation in transcription, mRNA stability, translation and post-translation levels.^156, 157, 158^ However, reports of the regulation of p53 by lncRNA currently are limited to post-transcriptional levels.^159^ Modulation of p53 by lncRNAs could be amplified by the aforementioned regulation networks, with profound effects in downstream apoptotic pathways. Wrap53*α* lncRNA is an endogenous antisense transcript of p53, which shares a 227bp overlap with p53 exon1 and stabilizes the p53 transcript through RNA–RNA interaction. Knockdown of Wrap53*α* abrogates p53-induced apoptosis, whereas overexpressing Wrap53*α* potentiates it.^160^ The maternally expressed gene 3 (MEG3) lncRNA utilizes its secondary motif M2 and M3 to activate p53,^161^ and selectively facilitates the activation of downstream p53-dependent apoptotic genes.^161, 162, 163^ The expression level of lncRNA MALAT1 is inversely correlated with that of p53, but without any proven structural evidence the correlation seems likely to be indirect.^164^ A particularly special p53-regulating lncRNA is ROR, which strongly suppresses p53 through formation of a complex with heterogeneous nuclear ribonucleoprotein I (hnRNP I). The expression of ROR itself is induced by p53, thus forming a negative feedback loop in control of p53 expression,^165, 166^ illustrating the complex nature of lncRNA–p53 interactions.

### Intrinsic apoptosis mediators regulated by lncRNA

INXS is an lncRNA transcribed from the opposite genomic strand of BCL-X, which shifts alternative splicing of BCL-X from the anti-apoptotic BCL-XL to the pro-apoptotic BCL-XS. Overexpression of INXS leads to accumulation of BCL-XS, activation of caspase-9 and -3, and subsequent apoptosis.^167^ PTEN, an indispensable mediator of STS-induced caspase-3 activation and cytochrome-c release,^168^ is subject to positive regulation by lncRNA PTEN pseudogene1 (PTENpg1), whereas PTENpg1 itself is regulated by two antisense transcript lncRNAs: PTENpg1 asRNA*α* and *β*. Specifically, the *α* isoform binds the PTEN promoter and inhibits transcription, whereas the *β* isoform stabilizes PTENpg1 and subsequently strengthens the upregulation of the PTEN gene.^169^ In addition, the aforementioned lncRNA MEG3 reduces BAX protein expression and caspase-3 activity, suppressing intrinsic apoptosis.^170^ Cancer cells could also deviate expression of intrinsic apoptosis-related lncRNAs to evade death. lncRNA-LET, for example, is downregulated by hypoxia in gallbladder cancer cells conferring apoptotic resistance, whereas ectopic expression of lncRNA-LET leads to an increased BAX/BCL-2 ratio, caspase-3 activation, and apoptosis.^171^ The crucial role of lncRNA in cancer is also highlighted by their relationships to some key oncogenes. Although tumorigenesis is typically attributed to multiple genetic abnormalities, it is shown to be dependent on one or a few key oncogenes, such as Myc, the deletion of which achieves a wide spectrum of anticancer effects, a phenomenon termed as ‘oncogene addiction'.^172, 173^ The lncRNAs that are regulated by Myc^174, 175^ may facilitate it in sustaining cancer cells, which makes them potential therapeutic targets, given the recent progress in nanoparticle-mediated miRNA delivery,^176^ corresponding therapeutic strategies may emerge in the foreseeable future.

### Extrinsic apoptosis mediators regulated by lncRNA

Mediators of the extrinsic apoptotic pathway, such as death-signal ligands, receptors and caspases, can be regulated directly or indirectly by lncRNAs. Soluble Fas (sFas), which sequesters FasL, is generated by RBM5-mediated alternative splicing of Fas mRNA that skips exon 6. The Fas antisense transcript lncRNA FAS-AS1 binds and inhibits RBM5 to inhibit sFas expression and strengthen Fas-FasL ligation.^177^ lncRNAs also target caspase-8, which is downstream of DR ligation, for example, lncRNA MALAT1 inhibits caspase-8 expression, which contributes to its anti-apoptotic function.^178^

### LncRNA and RN including necroptosis

The potential relationship of lncRNA to the regulation of necroptosis and other forms of RN is largely undefined at this time. Many lncRNAs have been predicted by computational scoring as necroptotic pathway regulators, and there is a commercially available microarray that integrates hundreds of these predicted lncRNAs. However, as of this writing, little experimental evidence has been published supporting such predictions.

## LncRNAs interact with miRNA in regulating cell death

**Figure 4:**
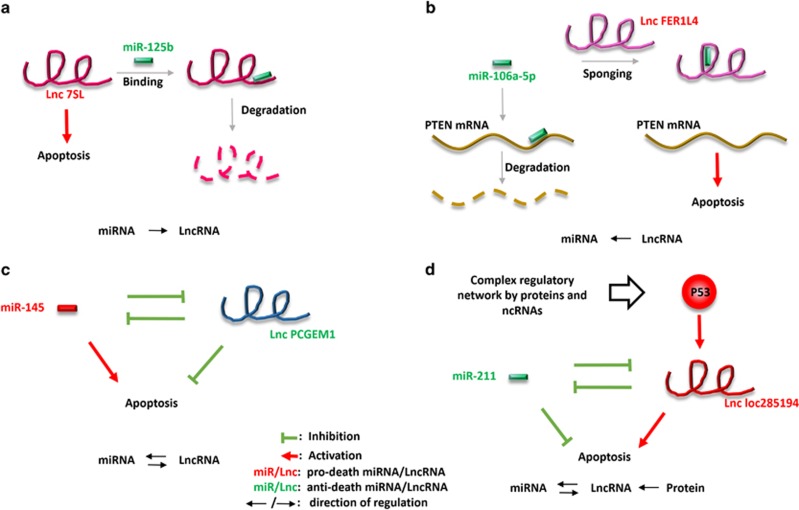
Four patterns of interactions between death-regulating miRNAs and LncRNAs. (**a**) An miRNA (miR-125b) inhibits an lncRNA (Lnc 7SL) from regulating cell death. (**b**) An lncRNA (Lnc FER1L4) works as a ceRNA to sponge up and sequester a miRNA (miR-106a-5p) from contacting and degrading death-related target mRNA (PTEN mRNA). (**c**) A given pair of death-regulating miRNA (miR-145) and lncRNA (Lnc PCGEM1) could suppress each other's expression and/or function. (**d**) A mutually suppressive pair of death-regulating miRNA (miR-211) and lncRNA (Lnc loc285194) could be subject to certain protein factor (P53)'s regulation

Theoretically, lncRNA and miRNA could interact with each other, adding an extra layer of complexity to the regulation of cell death by this class of RNA. In a transcriptome-wide bioinformatics analysis with several available databases, Jalali *et* *al.*^179^ recognized extensive interactions between numerous lncRNA–miRNA pairs. Specifically, many lncRNAs feature miRNA recognition elements preferentially clustered in their mid-to-3' region, which makes them potential targets of cognate miRNAs. On the other hand, some lncRNAs harbor miRNA regulatory elements with which they may target miRNAs. This study provided structural evidence for miRNA–lncRNA interactions, and highlighted the potential of bioinformatics in regulatory RNA studies. Indeed several miRNAs have consistently been reported to regulate lncRNA. For example, miR-125b, which binds and degrades p53 mRNA, has been found to target lncRNA 7sl as well (Figure 4a). This may also contribute to its anti-apoptotic function.^179^ Conversely, some lncRNAs have been shown to modulate miRNA function or expression, in at least two patterns. First, lncRNAs may sponge up and sequester miRNAs, in which case they work as a ceRNA to prevent the miRNA–mRNA contact,^125, 180^ for example, lncRNA FER1L4 and LncRNA RB1 are a pair of ceRNAs that both bind and sequester miR-106a-5p from targeting PTEN^181^ (Figure 4b). It is noteworthy, however, that a mere increase in miRNA target abundancy does not affect miRNA function,^182^ which means despite its name, ceRNA (including lncRNA) work by not only simply competing for miRNA binding, but may rely on spatial occlusion to block miRNAs from reaching their targets in the first place. Second, lncRNAs may suppress miRNA expression, as can be demonstrated by the example of lnc HULC, which suppresses the expression of the CREB-targeting miR-372 in HCC, and therefore affects CREB-mediated epigenetic modifications that govern the expression of a series of cell death/survival related genes.^183^ The regulatory relationship between a given pair of lncRNA–miRNA may be not only unidirectional but also bidirectional, which further interweaves the regulatory network among lncRNA, miRNA and cell death. For example, anti-apoptotic lncRNA PCGEM1 and pro-apoptotic miRNA miR-145 not only antagonize each other in regulating apoptosis but also suppress each other's expression, thus forming a regulation loop that regulates seemingly opposite effects on apoptosis^184^ (Figure 4c).

LncRNA–miRNA interactions are often dictated by protein factors and thus is integrated as a foundational element within an extensive cell death regulation network. As an upstream cell death modulator, p53's extensive effects on cell death is at least partially exerted through such lncRNA–miRNA interactions. Specifically, p53 targets a p53-response element within the upstream region of a pro-apoptotic lncRNA, loc285194, and induces its expression. A reciprocal repression has been shown to exist between loc285194 and the anti-apoptotic miRNA miR-211. Hence, the two regulatory ncRNAs and their intertwined effects on cell death are incorporated into an even more complicated p53 signaling network by the p53–lncRNA connection^185^ (Figure 4d). Even some of the protein-encoding mRNAs may exert an additional non-coding regulatory effect by competing with other mRNAs or lncRNAs for miRNA binding, and therefore also act as ceRNAs.^186^ This potential mechanism remains to be confirmed for death-related mRNAs.

In summary, ncRNAs are currently somewhat analogous to invisible dark matter in the universe. Just as dark matter accounts for >80% of universal mass and fundamentally affects the visible universe, the regulatory ncRNAs, despite being ‘invisible' in terms of encoded protein, comprise over 80% of total mature RNA and have many crucial but as yet, undefined roles in regulating programmed cell death. The two major subtypes of regulatory ncRNAs, miRNA and lncRNA interact with not only each other but also various intracellular components to extensively modulate the inter-related steps and mediators of regulated forms of cell death including apoptosis and necrosis. Thus, they may be in a pivotal position to regulate cell death. Although the interactions of the various components of regulatory ncRNA are incredibly complex and interactions will require considerable work to unravel, death-regulating ncRNAs may represent hugely important but as yet underutilized therapeutic targets in the manipulation of cell death and organ injury in diverse inflammatory clinical conditions.

## Figures and Tables

**Table 1:** miRNAs that regulate apoptosis or necroptosis

Target molecule	Regulatory molecule	Regulatory effect	Model	Source
*Intrinsic apoptosis*				
Bim	*miR-20 *miR-92 *miR-302 †miR-24 ‡miR-17-92a	Anti-apoptotic	*Mammalian pleuripotent stem cells †Cardiomyocytes ‡Osteoblasts	*Pernaute *et al.*^69^ †Wang and Qian^63^ ‡Guo *et al.*^71^
BCL2	*miR-15 *miR-16 †miR-24 †miR-195 †miR-365-2 ‡miR-181b/d	Pro-apoptotic	*Chronic lymphocytic leukemia †MCF7 breast cancer cells ‡MDR cell lines, glioma	*Cimmino *et al.*^48^ †Singh and * Saini^49^ ‡Zhu *et al.,*^51^ Wang *et al.*^52^
Bax	*miR-1 *miR-21 †miR-22 ‡miR-23a ‡miR-27a Lnc MEG	Anti-apoptotic Pro-apoptotic	*,†Myocardial IRI ‡Traumatic brain injury Trophoblast cells	*Duan *et al.*^61^ † Yang *et al.*^62^ ‡Sabirzhanov *et al.*^64^ Zhang *et al.*^161^
BCL-x	Lnc INXS	Pro/anti-apoptotic	Tumor tissue	DeOcesano-Pereira *et al.*^166^
P53	*Lnc Wrap53*α* †Lnc MEG3 ‡Lnc GAS5 *Lnc Malat1 †Lnc ROR	Pro-apoptotic Anti-apoptotic	*U2OS & HCT116 cells ‡Pkrostate cancer *Cancer cell lines †Stem cells	*Mahmoudi *et al.*^159^ †^159, 160, 161^ ‡Pickard^138^ Tripathi^163^ †Zhang *et al.,*^158^ Loewer *et al.*^165^
Par-4	T-ALL-R-LncR1	Anti-apoptotic	T-cell acute lymphoblastic leukemia	Zhang, Xu and Lu^129^
P38*α*	LncSPRY4-IT1	Anti-apoptotic	WM1552C melanoma	Khaitan *et al.*^132^
Wnt6	Lnc UCA1	Anti-apoptotic	Bladder cancer	Fan *et al.*^126^
APAF-1	miR-23a-27a-24 miR-23b-27b-24	Anti-apoptotic	Hypoxia-induced neuronal apoptosis	Chen *et al.*^75^
Caspase-9	miR-133	Anti-apoptotic	Myocardial preconditioning	He. *et al.*^76^
XIAP	*miR-23a †miR-24 ‡miR-130 miR-200bc-429	Pro-apoptotic	*Cerebral ischemia †Cancer cells ‡Ovarian cancer Gastric/lung cancer	*Siegel *et al.*^80^ †Xie *et al.*^81^ ‡Zhang *et al.*^82^ Zhu *et al.*^83^
PTEN	Lnc PTENpg1	Pro-apoptotic	Human cell lines	Johnsson *et al.*^168^
Caspase-3	miR-378 Lnc HIF 1 alpha-antisense RNA 1	Anti-apoptotic Pro-apoptotic	Cardiac myocytes Vascular smooth muscle cells	Fang *et al.*^77^ Wang *et al.*^137^
				
*Extrinsic apoptosis*				
Fas, TNF*α*	*miR-21 †miR-375 Lnc FAS-AS1	Anti-necro/apoptotic Pro-necro/apoptotic	*Cardiomyocytes, neurons, hepatocytes †Squamous cell carcinoma B-cell lymphoma	*^84, 85, 86^ †Wang *et al.*^88^ Sehgal *et al.*^176^
FADD	miR-27a miR-23a-27a-24	Pro-necro/apoptotic	Human embryonic kidney cells	Chhabra *et al.*^87^
Caspase-8	miR-874 lnc HOXA-AS2 lnc GAS5	Pro-necro/apoptotic Anti-necro/apoptotic Pro-necro/apoptotic	Myocardial cells Promelocytic Leukemia *Cancer cell lines	Wang *et al.*^95^ Zhao *et al.*^131^ *^138, 139, 140^
				
*Necroptosis*				
RIPK1	miR-155	Anti-necroptotic	Cadiomyocyte progenitor cells	Liu *et al.*^92^

Important miRNA regulators are enlisted along with their target molecules and the biological models they were studied upon.

## Abbreviations

ncRNA: non-coding RNA
miRNA: micro RNA
LncRNA: long non-coding RNA
UTR: untranslated region
MOMP: mitochondria outer membrane permeabilization
BCL2: B-cell lymphoma 2
BAX: Bcl-2-associated X protein
BAK: Bcl-2 homologous antagonist/killer
FasL: Fas ligand
